# The Diagnostic Journey from Rhabdomyolysis to Myopathy with Tubular Aggregates: A Family-Based Case Report and Review of the Literature

**DOI:** 10.3390/pediatric18040106

**Published:** 2026-08-05

**Authors:** Slavica Ostojić, Sanja Milenković, Sonja Pavlović, Gordana Kovačević, Gordana Petrović, Aleksandra Paripović, Adrijan Sarajlija, Marina Anđelković, Vladimir Gašić, Danijela Radivojević

**Affiliations:** 1 Department of Neurology, Mother and Child Health Care Institute of Serbia “Dr Vukan Čupić”, 11070 Belgrade, Serbia; gordana.kovacevic@imd.org.rs; 2 Faculty of Medicine, University of Belgrade, 11000 Belgrade, Serbia; aleksandra.paripovic@imd.org.rs (A.P.); adrijan.sarajlija@imd.org.rs (A.S.); 3 National Reference Laboratory for Neuromuscular Biopsies, University Clinical Hospital Center Zemun, 11080 Belgrade, Serbia; sanjamilenkovic3@gmail.com; 4 Department of Pathology, University Clinical Hospital Center Zemun, 11080 Belgrade, Serbia; 5 Institute of Molecular Genetics and Genetic Engineering, University of Belgrade, 11042 Belgrade, Serbia; sonja.pavlovic99@gmail.com (S.P.); marina.andjelkovic@imgge.bg.ac.rs (M.A.); vladimir.gasic@imgge.bg.ac.rs (V.G.); 6 Immunology Department, Mother and Child Health Care Institute of Serbia “Dr Vukan Čupić”, 11070 Belgrade, Serbia; gordana.petrovic@imd.org.rs; 7 Department of Nephrology, Mother and Child Health Care Institute of Serbia “Dr Vukan Čupić”, 11070 Belgrade, Serbia; 8 Division of Clinical Genetics, Mother and Child Health Care Institute of Serbia “Dr Vukan Čupić”, 11070 Belgrade, Serbia; 9 Laboratory of Medical Genetic, Mother and Child Health Care Institute of Serbia “Dr Vukan Čupić”, 11070 Belgrade, Serbia; daca72radivojevic@gmail.com

**Keywords:** myopathy, tubular aggregates, inflammatory myopathy, rhabdomyolysis, genome sequencing

## Abstract

**Introduction/Aims:** Myopathies with Tubular Aggregates (TAM) are rare, chronic neuromuscular disorders that may be inherited or acquired. The aim of this report is to present the diagnostic pathway and the challenges encountered in a family with three members affected by TAM caused by a rare *ORAI1* variant. **Case report**: Two siblings (15 and 11 years old) developed severe rhabdomyolysis triggered by a viral respiratory infection. Histopathological analysis demonstrated numerous tubular aggregates with mild focal secondary inflammatory changes and no immunophenotypic evidence of autoimmune inflammatory myopathy. Whole-exome sequencing identified a likely pathogenic heterozygous missense variant, NM_032790.3(ORAI1):c.319G>A (p.Val107Met), in the *ORAI1* gene, in both children and their asymptomatic mother. **Conclusions:** The identification of a rare *ORAI1* variant in this family supports the association with TAM, broadens the spectrum of phenotypic presentation, and illustrates the phenotypic variability that may exist even among affected members of the same family. Careful interpretation of inflammatory changes in muscle biopsy, together with immunohistochemical and genetic findings, is essential to avoid misclassification of hereditary tubular aggregate myopathy as autoimmune inflammatory myopathy.

## 1. Introduction

Myopathies with Tubular Aggregates (TAM) are rare, chronic neuromuscular disorders that may be either inherited or acquired and are characterized by the presence of tubular aggregates (TAs) within muscle fibers. These characteristic structures can be identified by routine histopathological examination and further characterized by electron microscopy [1]. These structures originate from the sarcoplasmic reticulum and are most associated with disturbances in calcium homeostasis within muscle cells. Recent findings suggest that the role of mitochondria in their formation cannot be excluded [2].

Clinically, TAM presents with progressive muscle weakness, typically affecting proximal muscles, and may manifest in either childhood or adulthood. Additional symptoms can include muscle cramps, pain, and occasionally mild myotonia [3,4]. Certain forms of TAM are linked to variants in genes such as *STIM1*, *ORAI1*, or *CASQ1*, and may follow either autosomal dominant or recessive inheritance patterns. Acquired forms have been associated with medication use or autoimmune conditions [1,3].

Tubular aggregates (TAs) are observed not only in TAM as a specific disorder, but also in skeletal muscle biopsies from individuals with other conditions, including periodic paralysis, paramyotonia congenita, congenital myasthenic syndromes, metabolic, toxic, and inflammatory myopathies, various familial myopathies, and myopathies of uncertain etiology [1,4]. They can also be present in skeletal muscles of some apparently asymptomatic individuals from ‘TAM’ families, who may therefore be clinically non-penetrant cases [1,3,5]. Ocular anomalies, such as ophthalmoplegia or pupillary abnormalities, may be present. In a few cases, central nervous system involvement has also been reported, manifesting as seizures or sensorineural hearing loss [4]. Diagnosis is based on clinical evaluation, electromyography (EMG), and histopathological (HP) analysis of muscle biopsy specimens. Electron microscopy can provide additional ultrastructural characterization of tubular aggregates in selected cases.

The purpose of this paper is to present the diagnostic pathway and the challenges encountered in a family with three members affected by TAM caused by a rare *ORAI1* variant, including an unusually severe episode of rhabdomyolysis.

## 2. Case Report

In March 2023, two biological siblings, a brother and sister, were admitted to the Department of Neurology at the Mother and Child Health Care Institute of Serbia “Dr Vukan Čupić.” The boy was 15 years and 5 months old, and the girl was 11 years old. The reason for admission was muscle weakness and pain, accompanied by elevated levels of muscle enzymes in the blood. Their parents are not consanguineous, and there is no family history of neuromuscular disorders. The personal medical history for both children is unremarkable. Both of the children are actively engaged in sports.

The onset of symptoms was preceded by a respiratory viral infection characterized by fever, nasopharyngitis, and subsequent muscle pain. All family members experienced similar respiratory symptoms; however, only the two affected children developed muscle pain and weakness. Neurological examination of both children at the time of admission revealed weakness predominantly affecting the shoulder and pelvic girdle muscles, the proximal muscles of the lower limbs, and the paravertebral musculature, graded 3–4/5 on the Medical Research Council (MRC) Manual Muscle Testing (MMT) scale [6]. Distal muscle strength was preserved (5/5) in both upper and lower extremities, except in the girl, who exhibited mild weakness of the wrist and ankle flexors and extensors (4/5). Overall, proximal muscle weakness was slightly more pronounced in the boy than in his sister. Deep tendon reflexes were preserved, except for diminished patellar reflexes in both patients. Both children had difficulty rising from a supine position, were using their elbows for support, and were unable to perform a full squat. Their gait was waddling, and both had difficulty walking on their heels due to bilateral Achilles tendon contractures. Calf muscle hypertrophy and shortened Achilles tendons were present in both children, as well as in their mother.

The children presented with severe rhabdomyolysis, as evidenced by markedly elevated serum muscle enzyme levels: for the boy creatine kinase (CK) was 65,055 U/L (reference value < 225 U/L), lactate dehydrogenase (LDH) 5229 U/L, aspartate aminotransferase (AST) 2032 U/L, and alanine aminotransferase (ALT) 908 U/L. For the girl, the results were: CK 25,947 U/L, LDH 4594 U/L, AST 637 U/L, and ALT 515 U/L. Urinalysis revealed proteinuria, myoglobinuria, and the presence of urate casts. Treatment included analgesic therapy (ibuprofen) and aggressive intravenous hydration combined with urine alkalinization and diuresis stimulation using furosemide. This approach led to a gradual decline in serum muscle enzyme levels, although complete normalization was not achieved during the three-week hospitalization period. A comprehensive nephrological evaluation was performed in the children, including renal ultrasound, assessment of renin and aldosterone levels, complement components (C3, C4), albuminuria screening, and 24 h ambulatory blood pressure monitoring. There was no evidence of renal pathology. Serum immunoglobulin levels were within the reference range in both patients. Antinuclear antibodies (ANA), perinuclear and cytoplasmic antineutrophil cytoplasmic antibodies (pANCA, cANCA), and anti–double-stranded DNA (anti-dsDNA) antibodies were all negative. The cardiological findings were normal. Electromyoneurography revealed normal nerve conduction studies and the presence of myopathic motor unit potentials in both proximal and distal limb muscles of both children. No spontaneous activity, including fibrillation potentials or positive sharp waves, was detected, and there were no electrophysiological features of an irritable myopathy suggestive of inflammatory muscle disease. Muscle magnetic resonance imaging (MRI) was not performed because of technical limitations at our institution. The children did not exhibit any clinical signs of central nervous system involvement.

Following hospital discharge, the children were referred for continued outpatient follow-up. They exhibited progressive clinical improvement over time, with increasing muscle strength and reduced fatigue during physical activity. In the meantime, we received the results of the myositis-specific antibody panel taken during hospitalization. The male patient tested positive for anti–PM-Scl75 and anti–EJ antibodies, while the female patient was positive for anti–Mi-2α antibodies. Nailfold capillaroscopy showed no abnormalities suggestive of dermatomyositis or other systemic autoimmune diseases. Given the persistently elevated CK levels, ongoing mild muscle weakness and pain, and a positive myositis-specific antibody panel, the decision was made to perform a muscle biopsy one month after the onset of symptoms. Immunosuppressive therapy was initiated after the biopsy. We decided also to perform whole exome sequencing (WES) to evaluate a suspected underlying hereditary myopathy shared by the siblings.

**Figure 1:**
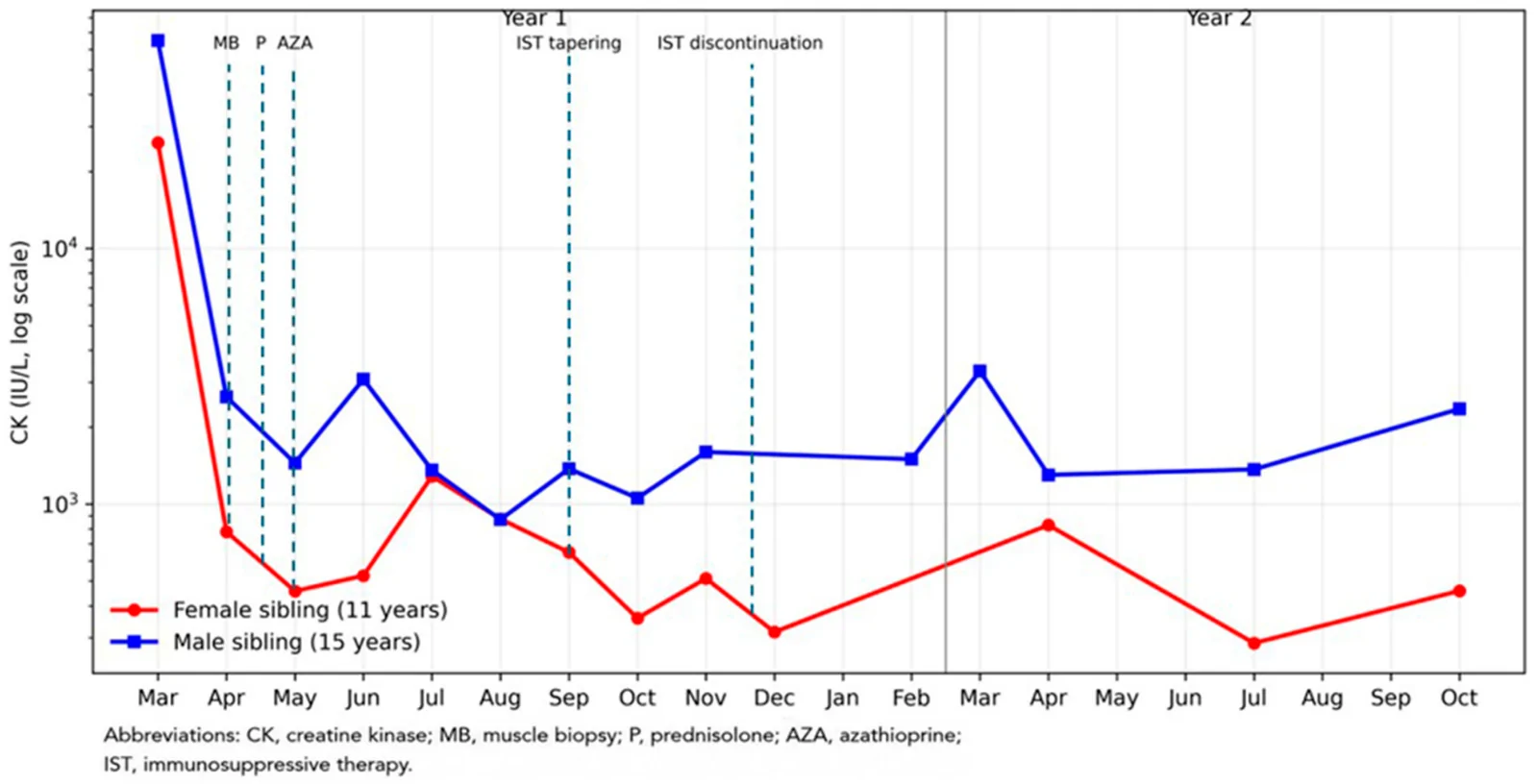
Serum creatine kinase (CK) levels in patients over time.

A three-day pulse therapy with methylprednisolone was administered, followed by oral prednisolone in tapering doses, and azathioprine as a steroid-sparing agent. Four months later, muscle biopsy results became available, revealing a myopathy characterized by tubular aggregates, most likely of hereditary origin rather than an inflammatory myopathy. The follow-up testing for myositis-specific antibodies was negative. We decided to gradually taper and ultimately discontinue immunosuppressive therapy. Muscle CK enzyme levels fluctuated over time, ranging from 870 to 2700 U/L in the boy and from 286 to 1300 U/L in the girl, without any signs of clinical deterioration. Figure 1 shows the fluctuations in creatine kinase levels in patients over time. A full clinical recovery was achieved.

### 2.1. Muscle Histopathological Findings

Quadriceps muscle biopsies were routinely analyzed by optical microscopy (Leica Microsystems GmbH, Wetzlar, Germany) [7]. Tissue fragments, frozen in liquid-nitrogen-cooled isopentane, were sectioned on a cryostat at 8–10 μm. Cryosections were stained with hematoxylin and eosin (H&E), modified Gomori trichrome, and enzyme histochemistry including NADH-tetrazolium reductase (NADH-TR), succinate dehydrogenase (SDH), cytochrome c oxidase (COX), combined COX/SDH, acid phosphatase, alkaline phosphatase, non-specific esterase, phosphorylase, phosphofructokinase, myoadenylate deaminase (MAD), and myofibrillar ATPase. Immunohistochemical (IHC) analyses were performed on formalin-fixed, paraffin-embedded tissue sections using antibodies against fast myosin, Ryanodine Receptor 1 (RyR1), dystrophin C, tropomyosin, myotilin, CD45 (LCA), CD4, CD8, CD68, MHC class I, and C5b-9. Sections were examined using a Leica DM2000 microscope equipped with a DFC295 digital camera(Leica Microsystems GmbH, Wetzlar, Germany) and LAS V4.7 software.

Histopathological examination demonstrated mild disturbance of the normal muscle architecture with variation in muscle fiber diameter, scattered atrophic fibers, occasional hypertrophic fibers without fiber splitting, and rare fibers undergoing myophagocytosis. More than 40% of muscle fibers contained finely granular basophilic material predominantly localized in the subsarcolemmal region, corresponding to tubular aggregates. Modified Gomori trichrome highlighted numerous subsarcolemmal and cytoplasmic tubular aggregates, whereas ragged-red fibers and inclusion bodies were absent. NADH-TR demonstrated intense enzymatic activity within the aggregates, while SDH and COX reactions showed altered oxidative enzyme activity with loss of enzymatic activity within the aggregates but without COX-negative fibers. Tubular aggregates were localized predominantly within type 2 muscle fibers. RyR1 and myotilin showed strong immunoreactivity within the aggregates, whereas tropomyosin expression was absent in these areas. Dystrophin C expression confirmed preserved sarcolemmal integrity.

Only mild focal inflammatory infiltrates were identified and did not represent the predominant histopathological finding. The inflammatory component was localized mainly in the perivascular connective tissue and in occasional foci of myophagocytosis. Immunohistochemistry demonstrated predominantly CD4-positive T lymphocytes, whereas CD8-positive T lymphocytes were extremely rare. CD68-positive macrophages were restricted to occasional foci of myophagocytosis. Importantly, MHC class I and C5b-9 immunostaining did not demonstrate an immunophenotype suggestive of autoimmune inflammatory myopathy.

**Figure 2:**
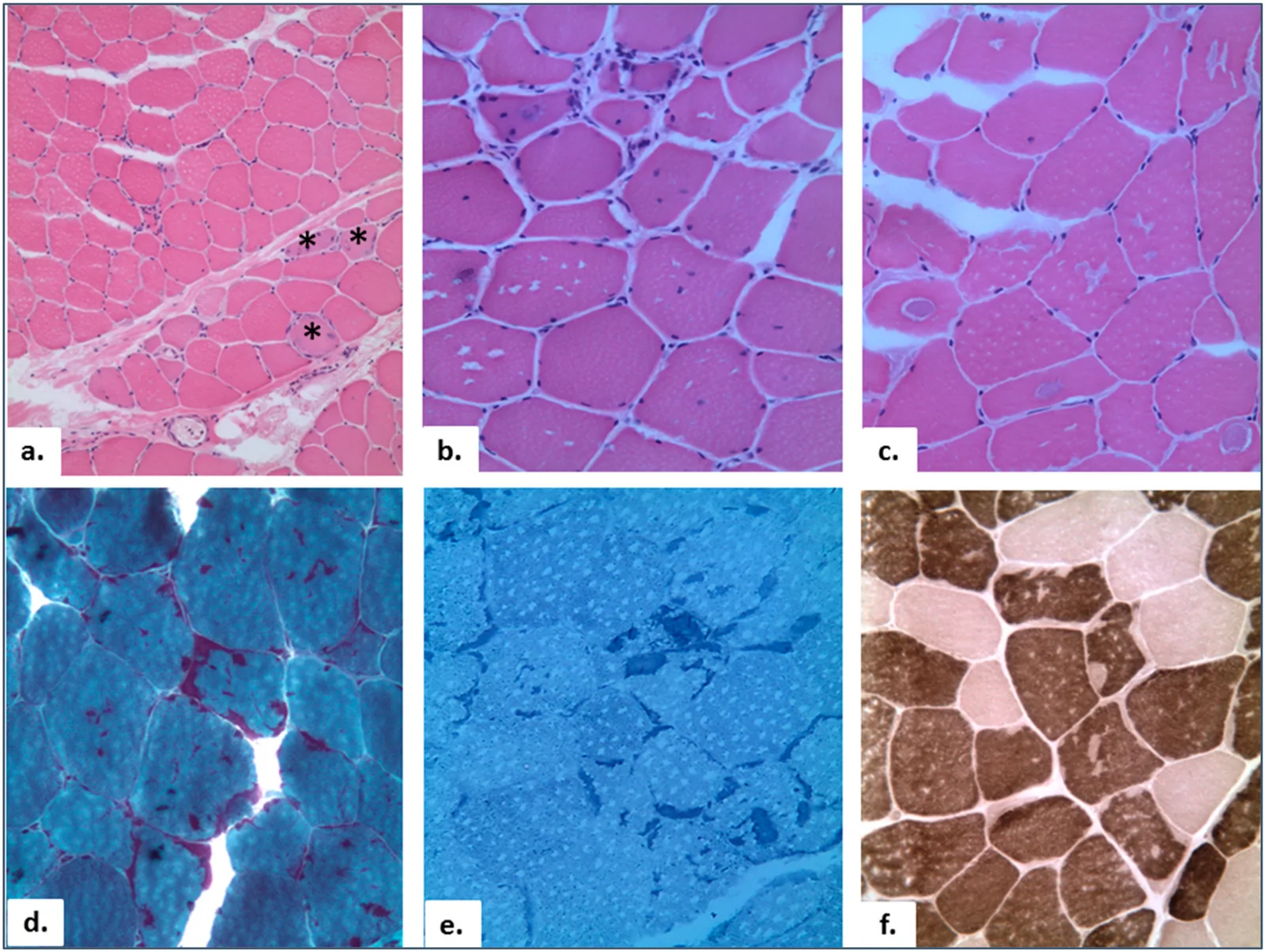
Representative histopathological findings in quadriceps muscle biopsies following H&E and histochemical staining. (**a**) Variation in muscle fiber diameter with scattered necrotic fibers (asterisks) (H&E, ×10). (**b**) Atrophic and centrally nucleated muscle fibers with focal myophagocytosis (H&E, ×10). (**c**) Numerous basophilic tubular aggregates with subsarcolemmal and internal localization (H&E, ×10). (**d**) Tubular aggregates stain bright red with modified Gomori trichrome (×10). (**e**) Tubular aggregates show intense NADH-tetrazolium reductase activity (NADH-TR, ×10). (**f**) Tubular aggregates are predominantly localized within type 2 muscle fibers (ATPase, pH 10.4, ×10).

**Figure 3:**
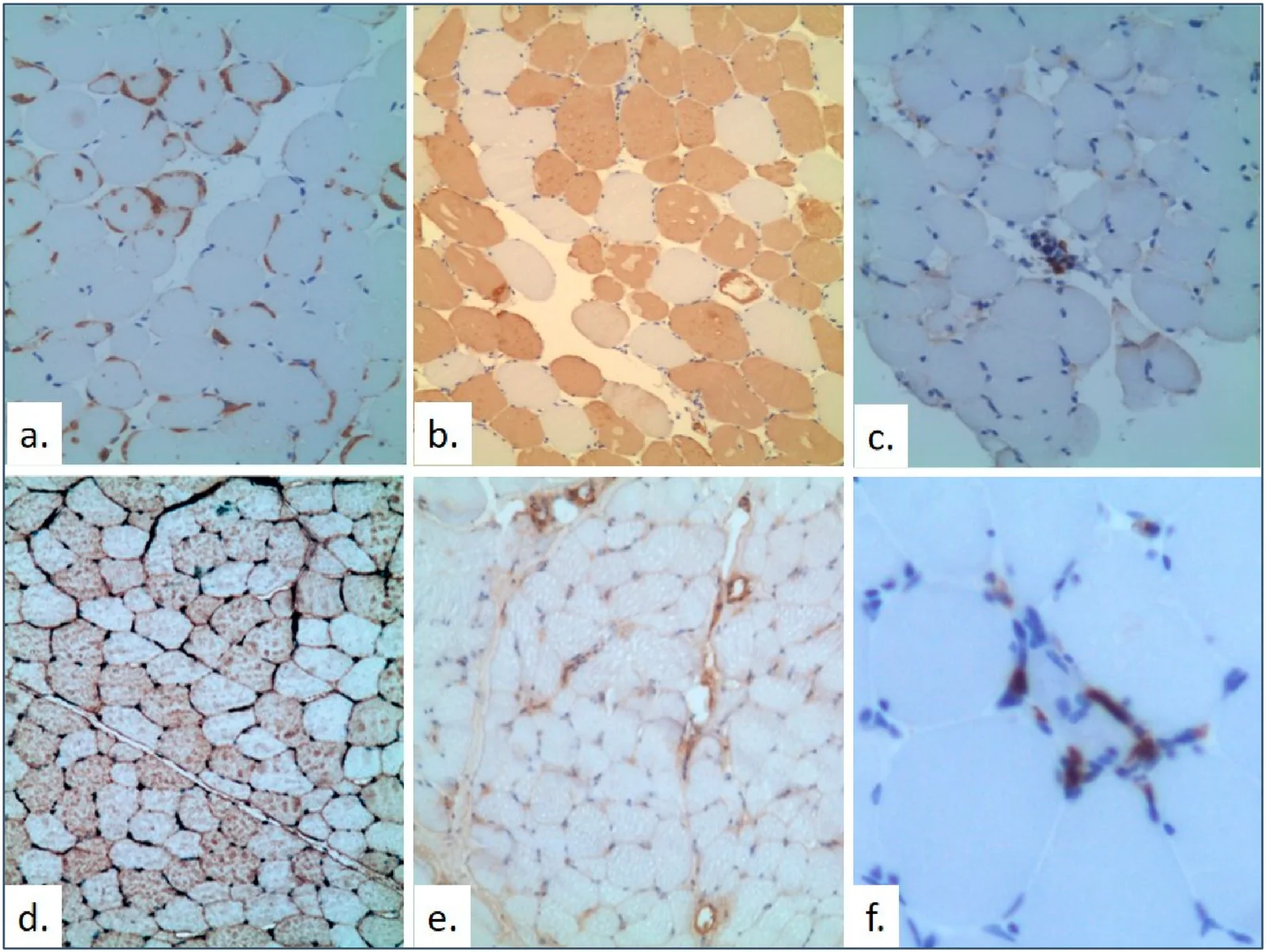
Findings from muscle biopsies of a brother and sister after applied imunohistochemical staining. (**a**) Ryanodine receptor 1 (RyR1) immunostaining demonstrates strong immunoreactivity within tubular aggregates (×10). (**b**) Fast myosin immunostaining demonstrates that tubular aggregates are predominantly localized within type 2 muscle fibers (×10). (**c**) CD68 immunostaining demonstrates CD68-positive macrophages restricted to occasional foci of myophagocytosis (×10). (**d**) MHC class I immunostaining shows no diffuse sarcolemmal expression in muscle fibers (×10). (**e**) C5b-9 immunostaining shows no abnormal sarcolemmal complement deposition in muscle fibers (×10). (**f**) CD4 immunostaining demonstrates sparse perivascular T-lymphocytic inflammatory infiltrates (×20).

Representative histopathological findings following H&E and histochemical staining are shown in Figure 2, whereas the immunohistochemical findings are shown in Figure 3.

### 2.2. Genetic Analysis

Genomic DNA was isolated from whole blood samples of the patients and relevant family members, using QIAamp DNA-Blood-Mini-Kit (QIAGEN, Hilden, Germany) and subsequently analyzed by Whole Exome Sequencing (WES). WES was performed using DNA Prep with Exome 2.5 Enrichment protocol (Illumina, San Diego, CA, USA). Analysis of sequencing by synthesis was performed on Illumina NextSeq 2000 platform (Illumina Inc., San Diego, CA, USA). Variants were annotated, filtered and classified using VarSome (Saphetor, Lausanne, Switzerland) for annotation and American College of Medical Genetics and Genomics (ACMG) guideline for variant classification [8,9].

Whole exome sequencing (WES) analysis revealed the presence of a heterozygous missense variant NM_032790.3(ORAI1): c.319G>A (pVal107Met) in the *ORAI1* gene (OMIM*610277, ORAI calcium release-activated calcium modulator 1) in both children [10]. According to one entry in the ClinVar classification, the variant is evaluated as pathogenic [11,12,13] and classified as likely pathogenic using ACMG guidelines. Data from the literature and population databases suggested that this variant is associated with tubular aggregate myopathy-2 (TAM2) (OMIM #615883, myopathy, tubular aggregate, 2; autosomal dominant inheritance) [14]. The same heterozygous variant was identified in their mother, who exhibited mildly elevated CK levels (400 U/L), but had no clinical signs of myopathy or history of rhabdomyolysis during infections. The variant was absent in their father, eldest sibling, and maternal grandfather.

## 3. Discussion

Tubular aggregate myopathy (TAM) is a rare congenital and acquired myopathy characterized by pronounced phenotypic heterogeneity, ranging from asymptomatic individuals to patients with progressive proximal muscle weakness, cramps, myalgia, and myotonia [1,2,3,4,5,11,12]. To our knowledge, this is among the very few reported pediatric/familial *ORAI1*-related TAM cases presenting with severe infection-triggered rhabdomyolysis as the dominant initial manifestation, with previously published isolated cases of TAM-associated rhabdomyolysis reporting significantly lower CK levels, including a conference presentation from São Paulo [15]. Our patients presented with the classic rhabdomyolysis triad: muscle weakness, myalgia, and dark urine. It is important to note that this triad is rarely fully expressed in pediatric patients, with <10% showing all three signs [16]. The risk of acute kidney injury (AKI) is a major concern in severe rhabdomyolysis. In our patients, despite the severity of muscle enzyme elevation, kidney complications were successfully prevented through prompt therapeutic intervention, consistent with current best practices [16]. While AKI occurs in up to 50% of adults with rhabdomyolysis, pediatric incidence is much lower (2.5–5%) [16].

Since two siblings presented with a highly similar clinical phenotype, an underlying hereditary myopathy was considered the primary working hypothesis from an early stage of the diagnostic work-up, while the episodes of rhabdomyolysis were interpreted as being triggered by viral infection in the context of the underlying genetic muscle disease. However, the diagnostic process in this family was particularly challenging because of the acute onset of muscle weakness and the transient presence of myositis-specific antibodies. Although an inherited myopathy remained the leading diagnostic consideration, these clinical and serological findings also raised concern for autoimmune inflammatory myopathy (AIM) and prompted the initiation of corticosteroid and azathioprine therapy. Importantly, although the muscle biopsy had already been performed before immunosuppressive therapy was initiated, the histopathological findings were not yet available when treatment decisions were made.

In retrospect, the transient antibody positivity was most likely nonspecific and associated with the preceding viral infection and severe muscle injury rather than representing a primary autoimmune process. Moreover, the detection of different myositis-specific antibodies in two siblings with an otherwise identical clinical phenotype further argues against AIM as the underlying diagnosis.

Histopathological analysis of the muscle biopsy played a crucial role in distinguishing hereditary myopathies from AIM [17]. Although mild inflammatory infiltrates were present, they required careful differential diagnostic interpretation because the transient myositis-specific antibodies initially suggested an autoimmune inflammatory process. However, the inflammatory infiltrates were focal and predominantly perivascular, while immunohistochemical analysis demonstrated no diffuse sarcolemmal MHC class I upregulation or C5b-9 deposition, findings that do not support immune-mediated muscle injury. Furthermore, CD68-positive macrophages were restricted to occasional foci of myophagocytosis. In contrast, the characteristic histochemical profile of tubular aggregates, their immunophenotype, and the identification of the same pathogenic *ORAI1* variant in both affected siblings and their clinically oligosymptomatic mother strongly supported hereditary tubular aggregate myopathy. Collectively, these findings indicate that the inflammatory infiltrates most likely represent secondary changes rather than evidence of concomitant autoimmune inflammatory myopathy. This interpretation is consistent with previous reports identifying hereditary myopathies as important mimics of idiopathic inflammatory myopathies [18]. It is possible that the administration of immunosuppressive therapy contributed to a somewhat faster clinical and laboratory recovery in the patients; however, they would likely have improved even without it, which is now evident.

In our experience, during respiratory virus seasons, particularly influenza and COVID-19 outbreaks, previously undiagnosed neuromuscular disorders may become clinically evident because of infection-triggered rhabdomyolysis. In addition to viral infections, several other factors such as trauma, fasting, excessive physical exercise, weight loss, alcohol intake, and febrile illness may trigger rhabdomyolysis in patients with congenital muscle disorders. These include muscular dystrophies, metabolic myopathies, and genetic defects of muscle metabolism (e.g., *ASO5*, *ACADVL*, *CPT2*, and *PYGM*), as well as disorders of intracellular calcium homeostasis (e.g., *RYR1*-related myopathies). [19]. Diagnosing these congenital conditions during episodes of rhabdomyolysis represents a particular challenge, given their rarity and phenotypic variability. Accurate diagnosis is crucial not only for determining disease prognosis, but also for providing preventive counseling aimed at avoiding future rhabdomyolysis episodes [19]. New diagnostic techniques such as NGS, WES and WGS may help evaluate patients to identify new genetic causes of rare inherited muscle diseases [19].

The heterozygous *ORAI1* c.319G>A (p.Val107Met) variant has previously been reported as a pathogenic gain-of-function variant causing autosomal dominant tubular aggregate myopathy (TAM2), and its pathogenicity has been confirmed by functional studies [11,12]. The previously reported patients exhibited a relatively consistent phenotype characterized by slowly progressive proximal muscle weakness, exercise intolerance, myalgia, muscle cramps, mildly elevated serum creatine kinase levels, myopathic electromyographic findings, and muscle biopsy demonstrating characteristic tubular aggregates [11]. The pathogenicity of the *ORAI1* p.Val107Met variant is further supported by functional studies demonstrating constitutive channel activation, altered channel gating, and increased store-operated calcium entry, consistent with a gain-of-function mechanism underlying tubular aggregate myopathy [12]. To date, other *ORAI1* pathogenic variants have also been reported in patients with isolated hyperCKemia, but in those cases, presenting as late-onset in the fifth or sixth decade of life [20], in contrast to our patients, who developed disease manifestations during adolescence. These observations further expand the clinical spectrum associated with the *ORAI1* p.Val107Met variant and provide additional evidence of its marked phenotypic variability.

One of the principal mechanisms responsible for maintaining intracellular Ca^2+^ homeostasis is store-operated calcium entry (SOCE), a highly regulated process mediated by the coordinated interaction between the sarcoplasmic/endoplasmic reticulum Ca^2+^ sensor STIM1 and the plasma membrane Ca^2+^ channel ORAI1 [21]. In skeletal muscle, SOCE is essential for calcium store refilling, sustained muscle contraction, and cellular adaptation to stress. Gain-of-function variants in either STIM1 or ORAI1 result in excessive intracellular Ca^2+^ influx, altered calcium signaling, sarcoplasmic reticulum remodeling, and ultimately tubular aggregate formation and muscle dysfunction [21,22,23]. 

Thus, although TAM is primarily considered a neuromuscular disorder, multisystem involvement has increasingly been recognized, particularly in patients with Stormorken syndrome [21,22]. In addition to progressive muscle weakness, cramps, myalgia, and exercise intolerance, affected individuals may present with thrombocytopenia, hyposplenism, miosis, short stature, ichthyosis, and central nervous system involvement [19,21,22]. TAM and Stormorken syndrome are now regarded as part of a continuous clinical spectrum rather than two distinct entities, with substantial phenotypic variability and incomplete genotype–phenotype correlation [22]. Inherited TAM is most commonly transmitted as an autosomal dominant disorder, although autosomal recessive forms have also been described [24]. Besides *STIM1* and *ORAI1*, pathogenic variants in several additional genes involved in calcium regulation and sarcoplasmic reticulum function have been associated with tubular aggregate myopathy, highlighting the marked genetic heterogeneity of this condition [4,24].

Genotype–phenotype correlations in *ORAI1*-related tubular aggregate myopathy remain incompletely understood, with substantial variability in disease severity, age at onset, degree of multisystem involvement, and histopathological findings. Earlier reports illustrating the phenotypic heterogeneity associated with *ORAI1*-related TAM are summarized in Table 1.

The main limitations of this study are the small number of related patients, the absence of functional validation of the identified variant, the lack of muscle MRI performed in a specialized pediatric neuromuscular imaging center, and the limited duration of follow-up, which precludes more definitive conclusions regarding disease course and phenotypic variability. Importantly, recent experimental studies have also identified ORAI1 inhibition as a potential therapeutic strategy, highlighting abnormal SOCE modulation as a promising future therapeutic target for TAM and related disorders [30].

## 4. Conclusions

In conclusion, this family case expands the clinical spectrum of *ORAI1*-related tubular aggregate myopathy and highlights severe infection-triggered rhabdomyolysis as a potential presenting manifestation of the disease. Our findings emphasize the diagnostic challenges posed by phenotypic overlap between congenital and autoimmune myopathies, particularly in pediatric patients presenting with acute muscle weakness and markedly elevated muscle enzymes. Careful interpretation of inflammatory changes in muscle biopsy, together with immunohistochemical and genetic findings, is essential to avoid misclassification of hereditary tubular aggregate myopathy as autoimmune inflammatory myopathy. In such cases, histopathological evaluation combined with comprehensive genetic testing is crucial for establishing an accurate diagnosis. Furthermore, growing insights into SOCE dysregulation and calcium homeostasis not only improve understanding of disease pathophysiology but may also facilitate the future development of targeted therapeutic approaches for TAM and related disorders.

## Figures and Tables

**Table 1:** Comparison of clinical and genetic features on *ORAI1* myopathy in previous studies.

Study No.	Authors (Year of Publication)	No. of Patients	Age at Onset	Clinical Presentation	Mode of Inheritance	Variant in *ORAI1* Gene
1	Shahrizaila et al. (2004) [25]	4	Late adult	Late adult-onset mild proximal muscle weakness with increased serum creatine kinase and areflexia of the lower limbs, Stormorken-like syndrome	AD, GoF	c.734C-T (P245L)
2	McCarl et al. (2009) [26]	6	Congenital childhood	Muscular hypotonia in all patients, Infections Autoimmunity, Ectodermal anomalies (all three genotypes), mydriasis	AR, AD,	c.271C>T (p.Arg91Trp) (2) p.Ala88SerfsX25 (1) c.308C>A (p.Ala103Glu)/ c.581T>C (p. Leu194Pro) (1) Not done in two
3	Endo Y et al. (2015) [27]	6 patients from 3 unrelated Japanese families	Childhood	Slowly progressive TAM with hypocalcemia	AD, GoF	c.292G-A, G98S; c.412C-T L138F
4	Lacruz and Feske review (2015) [28]	1	<1	Infections Autoimmunity		c.271C>T (p.Arg91Trp)
5	Garibaldi M. et al. (2017) [20]	3 patients from one Italian family	>50 y	A mild, late onset TAM revealed by asymptomatic CK elevation, muscle cramps during physical activity, mild symmetrical weakness and congenital miosis	AD, GoF	c.290C>G (p.S97C)
6	Böhm et al. (2017) [11]	9 patients from 3 unrelated families	From childhood to adult period	Proximal and distal weakness of lower limbs, myalgia, cramps, stiffness, contractures, Stormorken syndrome	GoF	G98S, V107M, T184M
7	Guillaume Baille et al. (2024) [4]	1	<1 y	Neonatal hypotonia, motor delay, a mild proximal and asymmetric muscular weakness, seizures, and sensorineural hearing loss	-	Genetic cause of TAM remains unsolved
8	Baskar D et al. (2024) [3]	1	15–16	Slowly progressive proximal lower limb weakness and ophthalmoparesis.	AR, LoF	c.205G>T (p.Glu69Ter)
9	Lian et al. (2019) [29]	6	<1	Congenital muscular hypotonia, Infections, Autoimmunity, CID Ectodermal anomalies, tooth defects, anhidrosis	AR, LoF	c.del541C (p.V181SfsX8), c.581T>C (p.L194P), c.G292C (p.G98R) C271T (p.R91W)

Abbreviations: No, number; CK, creatine kinase; AD, autosomal dominant; AR, autosomal recessive; GoF, gain-of-function; LoF, loss-of-function or null variants; CID, combined Immunodeficiency.

## Data Availability

All relevant data are contained within the manuscript.

## Abbreviations

The following abbreviations are used in this manuscript:

CASQ1	calsequestrin-1 gene
CACNA1S	calcium voltage-gated channel subunit alpha1S gene
CFTD	congenital fiber-type disproportion
CK	creatine kinase
EMG	electromyography
HP	histopathological
MMT	manual muscle testing
MRI	magnetic resonance imaging
NADH-TR	NADH-tetrazolium reductase
NGS	next-generation sequencing
ORAI1 (CRAMC1)	calcium release-activated calcium channel protein1 coding gene
RYR1	ryanodine receptor 1 coding gene
SOCE	store-operated calcium entry
STIM1	stromal interaction molecule 1 coding gene
TA	tubular aggregates
TAM	tubular aggregate myopathy
WES	whole-exome sequencing
WGS	whole-genome sequencing
